# In Memoriam

**Published:** 1889-06

**Authors:** Virgil O. Hardon, Wm. Perrin Nicolson, W. D. Bizzell


### IN MEMORIAM.

### ACTION OF THE ATLANTA SOCIETY OF MEDICINE.

At a meeting of the Atlanta Society of Medicine, held May 21, 1889, the following resolutions were unanimously adopted:

WHEREAS, It has pleased an All-wise Providence to remove from among us by death, our friend and fellow-member, Dr. A. B. Ashworth; therefore be it

*Resolved*, That in the death of Dr. Ashworth, this Society has lost an honored and valued member who has always shown, by word and deed, his interest in its welfare and success, and who has done valuable work in preserving the fruits of the labors of the Society and in giving them to the profession at large.

*Resolved*, That we extend to the bereaved relatives of the deceased our heartiest sympathy and condolence, and our appreciation of the severity of the affliction under which they are suffering.

*Resolved*, That these resolutions be spread upon the minutes of this society; that a copy be sent to the family of Dr. Ashworth, and that they be published in the ATLANTA MEDICAL AND SURGICAL JOURNAL, *The Atlanta Evening Journal* and *The Atlanta Constitution*.

VIRGIL O. HARDON,
WM. PERRIN NICOLSON,
W. D. BIZZELL.

